# The Application of Baum-Welch Algorithm in Multistep Attack

**DOI:** 10.1155/2014/374260

**Published:** 2014-05-28

**Authors:** Yanxue Zhang, Dongmei Zhao, Jinxing Liu

**Affiliations:** ^1^College of Mathematics and Information Science, Hebei Normal University, Shijiazhuang 050000, China; ^2^College of Information Technology, Hebei Normal University, Shijiazhuang 050000, China; ^3^The First Aeronautics College of PLAAF, Xinyang 464000, China

## Abstract

The biggest difficulty of hidden Markov model applied to multistep attack is the determination of observations. Now the research of the determination of observations is still lacking, and it shows a certain degree of subjectivity. In this regard, we integrate the attack intentions and hidden Markov model (HMM) and support a method to forecasting multistep attack based on hidden Markov model. Firstly, we train the existing hidden Markov model(s) by the Baum-Welch algorithm of HMM. Then we recognize the alert belonging to attack scenarios with the Forward algorithm of HMM. Finally, we forecast the next possible attack sequence with the Viterbi algorithm of HMM. The results of simulation experiments show that the hidden Markov models which have been trained are better than the untrained in recognition and prediction.

## 1. Introduction

Currently, the network security situation is increasingly sophisticated and the multistep network attack has become the mainstream of network attack. 2012 Chinese Internet network security reports released by the National Computer Network Emergency Response Technical Team Coordination Center of China (CNCERT/CC) show that the two typical multistep attacks: warms and distributed denial of service (DDOS) [1] account for 60% of overall network attacks. Multistep attack [2] means that the attacks apply multiple attack steps to attack the security holes of the target itself and achieve the devastating blow to the target. There are three features of attack steps of multistep attack. (1) In the multistep attack, there is a casual relationship between multiple attack steps. (2) The attack steps of multistep attack have the property of time sequence [3]. (3) The attack steps of multistep attack have the characteristics of uncertainty [4].

Multistep attack is one of the main forms of network attack behaviors, recognizing and predicting multistep attack that laid the foundation of active defense, which is still one of the hot spots nowadays. Literature (application of hidden Markov models to detect multistep network attacks) proposed a method to recognize multistep attack based on hidden Markov model.

Markov model literature (improving the quality of alerts and predicting intruder's next goal with hidden colored Petri-net) introduced the concept of attack “observation,” but both stayed in the specific attack behaviors, which have some limitations. Current research on the approaches to forecast multistep attack behaviors mainly includes four types: (1) the approach to forecasting multistep attack based on the antecedents and consequences of the attack [5]. It applies the precursor subsequent relationship of the event, to forecast the attacker wants to implement attacks in the near future. Because of the complexity and the diversity of the attack behaviors, this approach is difficult to achieve. (2) The approach to forecasting multistep attack based on hierarchical colored Petri-nets (HCPN) applies the raw alerts by Petri-nets and considers that the attack intention is inferred by raw alerts [4]. But this approach focuses on the intrusion detection of multistep attack behaviors. (3) The approach to forecasting multistep attack based on Bayes game theory could forecast the probability that the attackers choose to attack and the probability that the defenders choose to defend in the next stage rationally [6, 7]. However, in current study, only two-person game model is established, so this approach has some limitations. (4) The approach to forecasting multistep attack based on attack intention [3, 8] uses extended-directed graph to describe the logical relationship between attack behaviors and forecasts the next stage by logical relationship. The shortcoming of this approach is that it is difficult to determine the matching degree of the multistep attack. At the same time, there exists a certain degree of subjectivity in recognizing and forecasting multistep attack. In this regard, we integrate the attack intentions and hidden Markov model and propose a method to forecast multistep attack based on hidden Markov model. Firstly, we train the existing hidden Markov model(s) by the Baum-Welch algorithm of HMM. Then we recognize the alert belonging to attack scenarios with the Forward algorithm of HMM. Finally, we forecast the next possible attack sequence with the Viterbi algorithm of HMM. Simulation experiments results show that the hidden Markov models which have been trained are better than the untrained in recognition and prediction.

## 2. Hidden Markov Model

Hidden Markov model was first proposed by Baum and Petrie in 1966. It is a statistical model, which is used to describe a Markov process which contains a hidden parameter [9]. The research object of this model is a data sequence; each value of this data sequence is called an observation. Hidden Markov model assumes that there still exists another sequence which hides behind this data sequence; the other sequence consists of a series of states. Each observation occurs in a state, the state cannot be observed directly, and the features of the state can only be inferred from the observations.

A complete hidden Markov model (HMM) is usually represented by a triple *λ* = (*A*, *B*, *π*), which includes the following five elements:

1. a finite state, which is represented by the set *S*, where *S* = {*s*_1_, *s*_2_,…, *s*_*N*_}  and, at time *t*, the state is denoted by *q*_*t*_;
2. the set of observations, which is represented by the set *O*, where *O* = {*o*_1_, *o*_2_,…, *o*_*T*_};
3. the state transition matrix, which is represented by the matrix *A*, where *a*_*ij*_ = *p*[*q*_*t*+1_ = *s*_*j*_ | *q*_*t*_ = *s*_*j*_] and 1 ≤ *i*, *j* ≤ *N*;
4. the probability distribution of matrix *A*, which is represented by the matrix *B*,  where *b*_*j*_(*k*) = *p*[*o*_*k*_ | *q*_*t*_ = *s*_*j*_] and 1 ≤ *j* ≤ *N*, 1 ≤ *k* ≤ *T*;
5. the set of initial state probability distribution of HMM, which is represented by the set *π*, where *π*_*i*_ = *p*[*q*_1_ = *s*_*i*_] and 1 ≤ *i* ≤ *N*.

**Figure 1:**
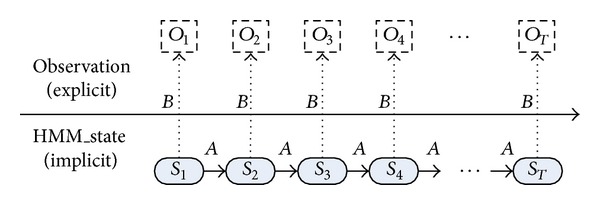
Model of recognizing and forecasting multistep attack based on hidden Markov model.

The model of recognizing and forecasting multistep attack based on hidden Markov model is shown in Figure 1.

There are three problems which can be solved by hidden Markov model well.

(1)* Probability Calculation Problems*. Calculate the probability *p*(*O* | *λ*) under a given hidden Markov model *λ* = (*A*, *B*, *π*) and the observation sequence *O* = {*o*_1_, *o*_2_,…, *o*_*T*_}.

(2)* Learning Problems*. Estimate the parameters of *λ* = (*A*, *B*, *π*) when the observation sequence *O* = {*o*_1_, *o*_2_,…, *o*_*T*_} is known, to maximize the probability *p*(*O* | *λ*).

(3)* Prediction Problems*. Calculate the state sequence *I* = {*i*_1_, *i*_2_,…, *i*_*T*_} under the maximum probability, when the hidden Markov model *λ* = (*A*, *B*, *π*) and observation sequence *O* = {*o*_1_, *o*_2_,…, *o*_*T*_} are given.

**Figure 2:**
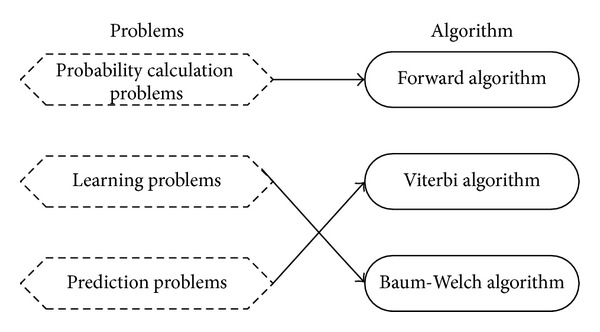
Correspondence between the problems and algorithms of hidden Markov model.

Correspondence between the problems and algorithms of hidden Markov model are shown in Figure 2.

Hidden Markov model is usually used to deal with the problems related to the time sequence and it has been widely used in speech recognition, signal processing, bioinformation, and other fields. Based on the characteristics of the attack steps of hidden Markov model and the problems that hidden Markov model can be solved, we apply the hidden Markov model to the field of recognizing and forecasting multistep attack. Firstly, the improved Baum-Welch algorithm is used to train the hidden Markov model *λ*, and we get a new hidden Markov model *λ*′. Then we recognize the alert belonging to attack scenarios with the Forward algorithm of hidden Markov model. Finally, we forecast the next possible attack sequence with the Viterbi algorithm of hidden Markov model.

## 3. The Approach to Recognizing and Forecasting Multistep Attack

The steps of the approach to recognizing and forecasting multistep attack are as follows.

**Step 1**

Obtain the initial state matrix (old), state transition matrix (old), and observation matrix (old) of HMM (*λ*).

**Step 2**

Use the improved Baum-Welch algorithm to train the initial state matrix (old) and observation matrix (old), and we get an initial state matrix (new), observation matrix (new), and a new HMM (*λ*′).

**Step 3**

Recognize the alert belonging to attack scenarios with the Forward algorithm.

**Step 4**

Forecast the next possible attack sequence with the Viterbi algorithm.

**Figure 3:**
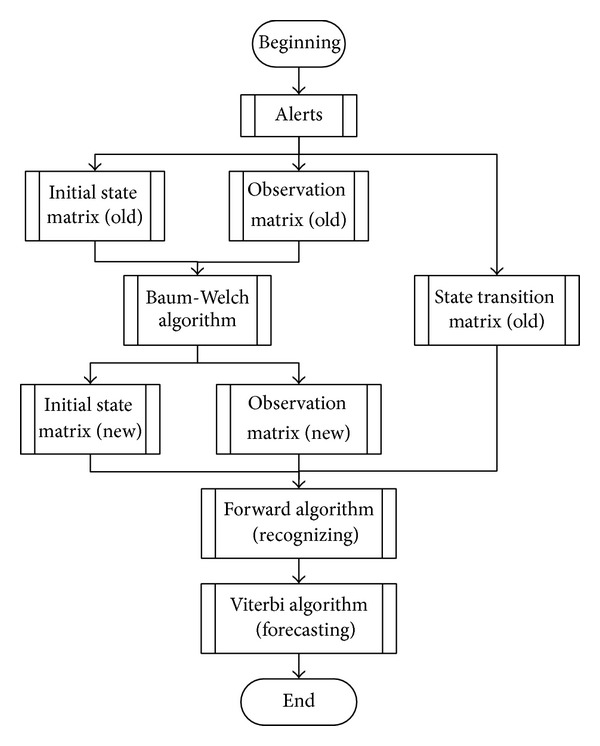
Flow chart of recognizing and forecasting multistep attack.

The flow chart is shown in Figure 3.

### 3.1. The Introduction of Baum-Welch Algorithm

If we want to apply the hidden Markov model to the multistep attack, the biggest problem is to determine the observations of HMM. A better parameter can improve the efficiency of calculation. Meanwhile, if the selection of observation is improper, this may result in a longer training time and even not complete the training. In this regard, we apply the Baum-Welch algorithm to train the given hidden Markov model. From the result of literature (accurate Baum-Welch algorithm free from overflow), we can learn that the most reliable algorithm to train the HMM is Baum-Welch algorithm. Baum-Welch algorithm can train the given hidden Markov model (*λ*) by an observation sequence and generate a new hidden Markov model (*λ*′) for detection.

The steps of Baum-Welch algorithm are as in Algorithm 1.

### 3.2. Forward Algorithm

The pseudocode of Forward algorithm is as in Algorithm 2.

**Figure 4:**
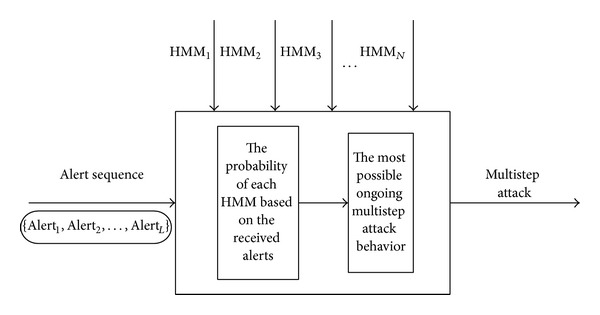
The structure of recognizing multistep attack with Forward algorithm.

Recognizing multistep attack is mainly based on the alert sequence. First, we calculate the probability of alert sequence generated by the given HMM(s). Then we decide that the attack which has the maximum is likely to be the ongoing attack. The structure of recognizing multistep attack with Forward algorithm is shown in Figure 4.

### 3.3. Viterbi Algorithm

The pseudocode of Viterbi algorithm is as in Algorithm 3.

**Figure 5:**
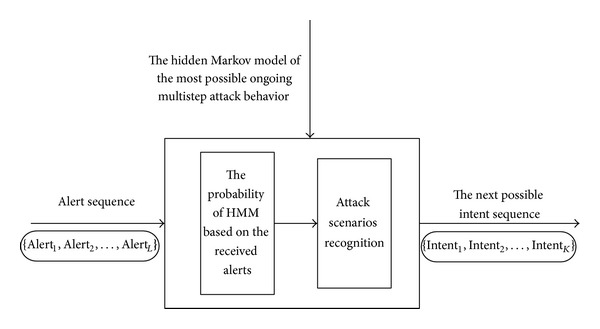
Forecasting multistep attack with Viterbi algorithm.

Predicting the behavior of multistep attack is mainly to determine the intentions that the attackers have been completed and forecast the next possible attack intentions. The structure of forecasting multistep attack with Viterbi algorithm is shown in Figure 5.

## 4. The Simulation Experiment and Analysis

### 4.1. Baum-Welch Algorithm: Train the Given HMM(s)

Based on the literature (approach to forecast multistep attack based on fuzzy hidden Markov model), we can obtain the initial state matrix, state transition matrix, and observation of DDoS_HMM, as is shown from Tables 1, 2, and 3.

The data set which is used in the simulation experiment is an attack scenario testing data set LLDOS1.0 (inside) provided by DARPA (Defense Advanced Research Projects Agency) in 2000. We extract two kinds of multistep attack from it; they are DDoS multistep attack and FTP Bounce multistep attack. While the calculation of the state transition matrix is completely the statistical calculations on data, we only train the initial state matrix and observation matrix of HMM. We can see that there are a large number of zeros in observation matrix clearly and the observation matrix is the sparse matrix. So we train the matrix(s) by block. We suppose that the number of observation sequences is *S* and the length of *S* is 32, where *S* multiplied by 32 equals the number of training data. And there is no corresponding sequence of state. In this regard, we can obtain the initial state matrix (new) and the observation matrix (new) of the DDoS_HMM′ (*λ*′), as is shown in Tables 4 and 5.

### 4.2. Forward Algorithm: Recognize the Alert Belonging to Attack Scenarios

The attack intentions and alerts of DDoS_HMM and FTP Bounce_HMM are shown in Tables 6 and 7, respectively.

When the alerts “Alert_1_” and “Alert_3_” were received, according to the Forward algorithm of hidden Markov model, we will obtain the probability based on DDoS_HMM′ and FTP Bounce_HMM′, respectively:

 
*p*(alerts | DDoS_HMM) = 0.2989,
 
*p*(alerts | FTP Bounce) = 0.0036.

We can see from the above results,* p*(alerts | DDoS_HMM) >* p*(alerts | FTP Bounce). That is to say, the ongoing multistep attack behavior is likely to be DDoS_HMM.

### 4.3. Viterbi Algorithm: Forecast the Next Possible Attack Sequence

When the alert sequence {Alert_1_, Alert_3_, Alert_7_, Alert_8_, Alert_10_} was received by the console, we can obtain the completed intent sequence {State_1_, State_2_, State_3_, State_4_}. That is to say, now completed intentions are the previous four attack intentions; the next intention will be state_5_.

### 4.4. Comparison of Results

We compare the results between the untrained HMM(s) and the trained HMM(s) by Baum-Welch algorithm; the comparison of results are shown in Table 8.

## 5. Conclusion

The biggest difficulty of hidden Markov model applied in multistep attack is the determination of observations. Now the research of the determination of observations is still lacking, and it shows a certain degree of subjectivity. In this regard, we train the existing hidden Markov model(s) by the Baum-Welch algorithm of HMM based on several groups of observation sequence. And we can obtain a new hidden Markov model which is more objectively. Simulation experiments results show that the hidden Markov models which have been trained are better than the untrained in recognition and prediction.

## Figures and Tables

**Algorithm 1:**
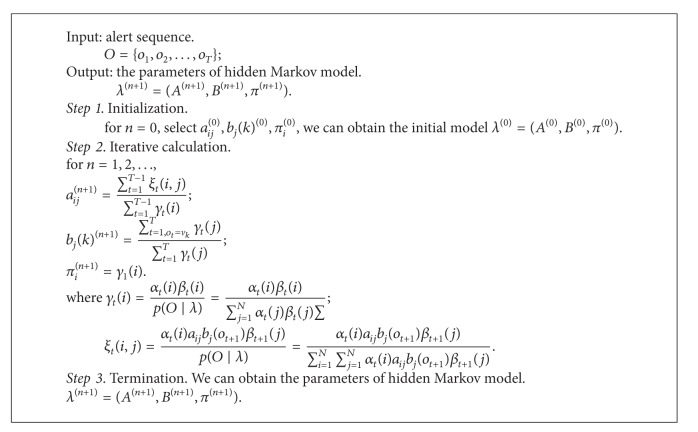
titleworktilte

**Algorithm 2:**
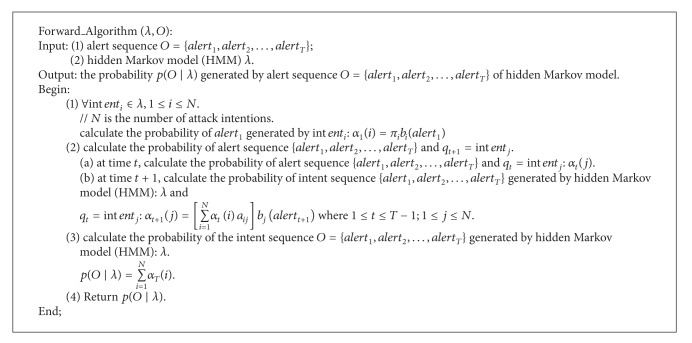


**Algorithm 3:**
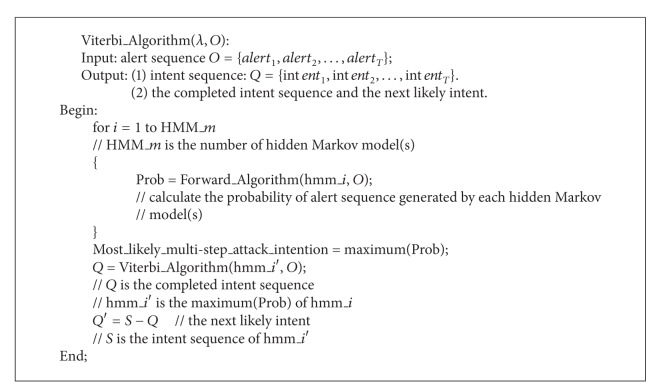


**Table 1:** The initial state matrix of DDoS_HMM.

State_1_	State_2_	State_3_	State_4_	State_5_
0.250	0.750	0.000	0.000	0.000

**Table 2:** The state transition matrix of DDoS_HMM.

	State_1_	State_2_	State_3_	State_4_	State_5_
State_1_	0.000	1.000	0.000	0.000	0.000
State_2_	0.000	0.177	0.823	0.000	0.000
State_3_	0.000	0.228	0.688	0.028	0.056
State_4_	0.000	0.000	0.000	0.750	0.250
State_5_	0.000	0.000	0.000	0.000	0.000

**Table 3:** The observation matrix of DDoS_HMM.

	*A*1	*A*2	*A*3	*A*4	*A*5	*A*6	*A*7	*A*8	*A*9	*A*10	*A*11	*A*12	*A*13
*S*1	1.000	0.000	0.000	0.000	0.000	0.000	0.000	0.000	0.000	0.000	0.000	0.000	0.000
*S*2	0.000	0.490	0.490	0.020	0.000	0.000	0.000	0.000	0.000	0.000	0.000	0.000	0.000
*S*3	0.000	0.000	0.000	0.000	0.200	0.200	0.200	0.200	0.200	0.000	0.000	0.000	0.000
*S*4	0.000	0.000	0.000	0.000	0.000	0.000	0.000	0.000	0.000	1.000	0.000	0.000	0.000
*S*5	0.000	0.000	0.000	0.000	0.000	0.000	0.000	0.000	0.000	0.000	0.660	0.170	0.170

**Table 4:** The initial state matrix of DDoS_HMM′.

State_1_	State_2_	State_3_	State_4_	State_5_
0.599	0.401	0.000	0.000	0.000

**Table 5:** The observation matrix of DDoS_HMM′.

	*A*1	*A*2	*A*3	*A*4	*A*5	*A*6	*A*7	*A*8	*A*9	*A*10	*A*11	*A*12	*A*13
*S*1	1.000	0.000	0.000	0.000	0.000	0.000	0.000	0.000	0.000	0.000	0.000	0.000	0.000
*S*2	0.000	0.499	0.499	0.002	0.000	0.000	0.000	0.000	0.000	0.000	0.000	0.000	0.000
*S*3	0.000	0.000	0.000	0.000	0.387	0.000	0.387	0.000	0.226	0.000	0.000	0.000	0.000
*S*4	0.000	0.000	0.000	0.000	0.000	0.000	0.000	0.000	0.000	1.000	0.000	0.000	0.000
*S*5	0.000	0.000	0.000	0.000	0.000	0.000	0.000	0.000	0.000	0.000	0.998	0.001	0.001

**Table 6:** DDoS_HMM.

STATE	ALERT
State_1_	{Alert_1_}
State_2_	{Alert_2_, Alert_3_, Alert_4_}
State_3_	{Alert_5_, Alert_6_, Alert_7_, Alert_8_, Alert_9_}
State_4_	{Alert_10_}
State_5_	{Alert_11_, Alert_12_, Alert_13_}

**Table 7:** FTP Bounce_HMM.

State	Alert
State_1_	{Alert_1_′, Alert_2_′}
State_2_	{Alert_3_′, Alert_4_′}
State_3_	{Alert_5_′, Alert_6_′, Alert_7_′}
State_4_	{Alert_8_′}
State_5_	{Alert_9_′, Alert_10_′}

**Table 8:** The comparison of results.

	*p*(alerts | DDoS_HMM)	*p*(alerts | FTP Bounce_HMM)	*p*(alerts | DDoS_HMM)
			*p*(alerts | FTP Bounce_HMM)
Before training	0.1225	0.0079	15.5
After training	0.2989	0.0036	83.0
